# Vertical Field Emission Air-Channel Diodes and Transistors

**DOI:** 10.3390/mi10120858

**Published:** 2019-12-06

**Authors:** Wen-Teng Chang, Hsu-Jung Hsu, Po-Heng Pao

**Affiliations:** Department of Electrical Engineering, National University of Kaohsiung, Kaohsiung 81148, Taiwan; asdf30103@gmail.com (H.-J.H.);

**Keywords:** vacuum channel, mean free path, vertical air-channel diode, vertical transistor, field emission, particle trajectory model, F–N plot, space-charge-limited currents

## Abstract

Vacuum channel transistors are potential candidates for low-loss and high-speed electronic devices beyond complementary metal-oxide-semiconductors (CMOS). When the nanoscale transport distance is smaller than the mean free path (MFP) in atmospheric pressure, a transistor can work in air owing to the immunity of carrier collision. The nature of a vacuum channel allows devices to function in a high-temperature radiation environment. This research intended to investigate gate location in a vertical vacuum channel transistor. The influence of scattering under different ambient pressure levels was evaluated using a transport distance of about 60 nm, around the range of MFP in air. The finite element model suggests that gate electrodes should be near emitters in vertical vacuum channel transistors because the electrodes exhibit high-drive currents and low-subthreshold swings. The particle trajectory model indicates that collected electron flow (electric current) performs like a typical metal oxide semiconductor field effect-transistor (MOSFET), and that gate voltage plays a role in enhancing emission electrons. The results of the measurement on vertical diodes show that current and voltage under reduced pressure and filled with CO_2_ are different from those under atmospheric pressure. This result implies that this design can be used for gas and pressure sensing.

## 1. Introduction

Several potential candidates for low-loss and high-speed electronic transistors beyond complementary metal oxide semiconductors (CMOS) have been proposed. Two-dimensional materials, such as graphene and transition metal dichalcogenide, were considered for next generation transistors [1,2,3]. Vacuum channel transistors have also been considered candidates because they are 10 times faster than silicon-based transistors [4]. The nature of vacuum channels enables the devices to function at elevated temperatures and radiation levels. A variety of structures in building vacuum channel transistors have been proposed in recent years [4,5,6,7,8,9,10,11,12,13,14], although vacuum transistors were first proposed to build circuits a few decades ago [15]. Generally, if the transport distance of a field emission (FE) is greater than the submicroscale, a vacuum condition is required to prevent carrier scattering, due to collision with moving particles in an ambient environment, and to achieve ballistic transport [16,17,18,19]. In addition, such a distance (or longer) generally requires a high driving voltage, which is impractical in large-scale integrated circuits [20]. Several studies have reported that FE devices with a transport distance as small as the nanoscale enable devices to operate at low voltages [5,6,21,22]. Therefore, reducing the transport distance to smaller than the mean free path (MFP) allows devices to operate in atmospheric pressure [5,6,7,11,12,13] and function under a small voltage that is suitable for practical circuits. The benefits of using 2D materials can be exploited with a vacuum channel device for low-leakage and high-speed applications [23]. 

Nanoscale FE air channel devices are generally categorized into horizontal, vertical, and all-around gate, depending on the related locations of emitter collectors and gates. Nanoscale horizontal electron emission air channel devices generally require advanced lithography technology or a trimming approach to define transport distance [4,5,6,7,10,11,12,13,14]. By contrast, vertical electron emission air channel devices define transport distance on the basis of the thicknesses of metal-dielectric stacked films [8,9]. Thin films stacked by atomic layer deposition provide an approach for specifying the transport distance of electron emission. The emission zone can be determined by the width of an open cavity on stacked films [7,8,9]. Gates in vacuum channel transistors are required for modulating tunneling current like in the conventional solid-state transistors. The bottom or surrounding gates of the horizontal and vertical FE transistors generally locate the gates between emitters and collectors so that the gate electric field can effectively modulate emission current [4,6,7,8,9,11,12,13,14,18]. However, several studies showed that an increase in the gate electric field can still influence the emission current when gate electrodes are above the route of electron emission [10]. The distance between the gate and the emitter can influence the turn-on voltage and controllability on the emission current for a microscale vacuum channel transistor [16,17,18,19]. However, the role of gates in vertical transistors has not been well studied. A nanoscale FE transistor can perform differently from a microscale FE one because electrons can be intercepted by coplanar gates under a nanoscale distance [13]. This paper used the finite element modeling to predict electron trajectory and determine the optimal gate location in a vertical vacuum channel transistor. Hence, carrier scattering in air, which may be difficult to find by the current simulation, must be considered in practical FE devices. The other aims of this paper were to fabricate a vertical diode with a transport distance near the MFP (~68 nm) in atmospheric pressure and to discuss carrier scattering under atmospheric and other ambient pressures.

## 2. Experiment

### 2.1. Modeling of Vertical Vacuum Channel Transistors

**Figure 1:**
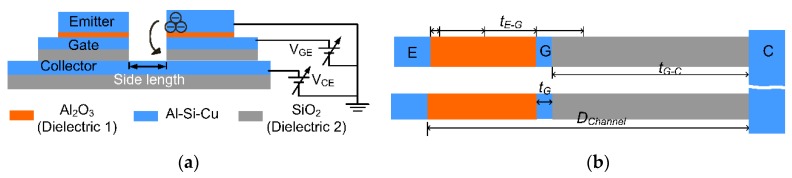
(**a**) Schematic of the vertical vacuum channel transistor composed of stacked layers of emitters (Al-Si-Cu), a gate dielectric (Al_2_O_3_), a gate (Al-Si-Cu), a dielectric (SiO_2_), and a bottom collector (Al-Si-Cu). (**b**) Transport distance of channel (D_Channel_) made up of the thicknesses of dielectric between the emitter and the gate (t_E-G_), gate (t_G_), and dielectric between the gate and the collector (t_G-C_) between the emitter (E) and the collector (C).

The schematic of a vertical vacuum channel transistor includes stacked layers of emitters, an emitter-gate dielectric (Dielectric 1), a gate, a gate-collector (Dielectric 2), and a bottom collector (Figure 1a). The etched well allows emission electrons to be ejected from the top emitter and collected by the bottom collector through the application of voltage potential (V_CE_). The application of gate potential (V_GC_) can modulate the route and the final speed of the electron emission. The side length of the square well is 2 μm, that is, the total emission width is four times the side width. The thicknesses of the emitter-to-gate (Dielectric 1), gate, and gate-to-collector (Dielectric 2) are t_E-G_, t_G_, and t_G-C_, respectively (Figure 1b). The four transistors with different t_E-G_ were investigated, namely, EG_3 (t_E-G_ = 3 nm), EG_10 (t_E-G_ = 10 nm), EG_20 (t_E-G_ = 20 nm), and EG_30 (t_E-G_ = 30 nm) (Table 1). The channels of transport distance (D_channel_) from emitter to collector are nearly the same (~60 nm). The modeling software COMSOL (5.2, COMSOL Inc., Stockholm, Sweden) is used to model particle trajectory and electric field. The particle trajectory model assumes only a 1 nm thickness window on the lower edge of the emitter because the edges of electrodes present the highest electric fields [24]. The window releases the finite electrons (400 particles) to graphically present tracing electrons. The collector counts the collected electrons with different V_CE_ and V_GC_. The Keithley 2400 current–voltage source meter is used for electrical characterization. The software by COMSOL is used to model the electric fields and particle trajectory in the vertical channel transistors. The material outside the device is air.

### 2.2. Fabricated Vertical Field Emission Diodes and Experimental Measurement

**Figure 2:**
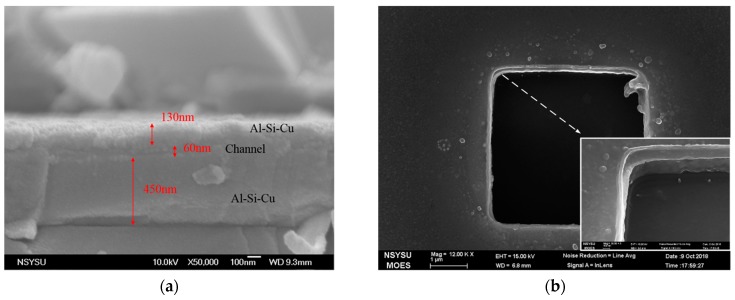
(**a**) Cross-sectional deposited stacked thin films, in which the thickness of the SiO2 (distance of electrons), the top (emitter), and the bottom (collector) Al-Si-Cu alloy are 60, 130, and 450 nm, respectively. (**b**) Top view of the micrograph of the etched cavity and tilt view (inset) showing the profile of the stacked thin films.

The fabrication of the vertical air channel diodes deposits 1 μm SiO_2_ for the isolation of devices from a Si p-type (111) substrate. The photo masks define the diode-probing areas of the collector and emitter made of an Al-Si-Cu alloy, in which Al accounts for 99.5% of the metal. The SiO_2_ thin film is sandwiched by the top and bottom Al-Si-Cu alloy layers. SiO_2_ thickness is roughly the transport distance of electron emission because the electric field is concentrated at the edge of the metal. The cross-section of the stacked thin films indicates that the thicknesses of the top and bottom Al-Si-Cu films are 130 nm and 450 nm, respectively (Figure 2a). Square wells of 2 μm × 2 μm and 4 μm × 4 μm are etched so that electrons are transmitted from the top emitter to the collector that is located on the bottom of the well (Figure 2b). The tilt view of the etched well presents the profiles of the Al-Si-Cu layers (inset of Figure 2b). The MFP is the average distance at which a particle travels between two successive collisions. Therefore, a SiO_2_ thickness of 60 nm is applied to enhance sensibility at varying ambient pressures. Transport distance in air is presumed to be approximately equal to the MFP.

**Figure 3:**
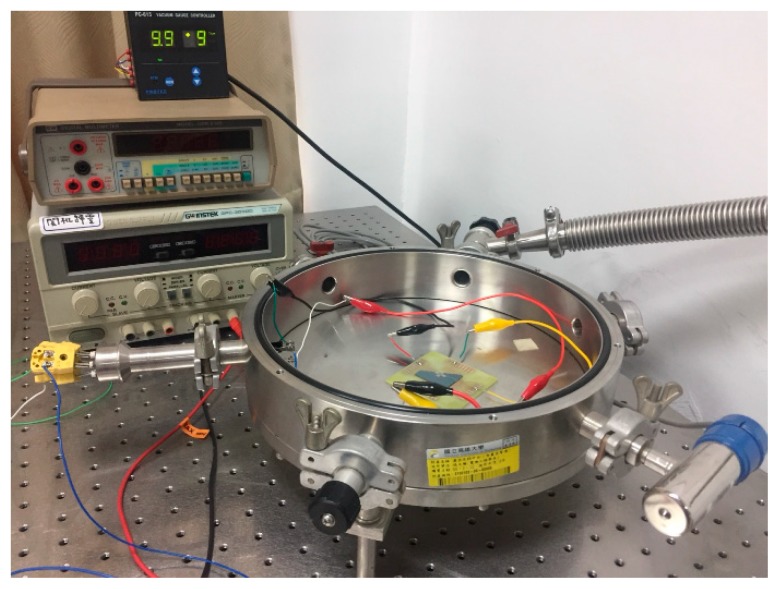
Customized vacuum chamber facilitated with a wire port, vacuum pump, gas inlet, pressure regulator, and pressure indicator used to measure the devices under different pressure levels.

The devices were wire-bonded on a printed circuit board and were measured in a customized vacuum chamber (Figure 3). The electric signals were connected to the measurement units outside the chamber via wire ports. The gas inlet of the chamber was connected to a gas feedthrough to allow external gases to flow into the chamber. A dry pump was attached to the chamber, allowing a minimum pressure of 10 mTorr.

## 3. Results and Discussion

### 3.1. Current Density Modeling of Field Emission Diodes

**Figure 4:**
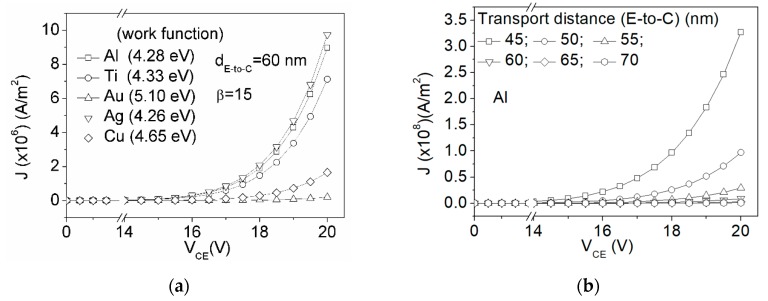
Electric current density (*J*) as a function of applied voltage (V_CE_) for a high electric field applying the Fowler–Nordheim equation for (**a**) metals that exhibit different work functions (*ϕ*) and (**b**) different transport distances by using Al metal.

Space charges under high and low electric fields differ owing to the polarity of space charges. Charges in the high field are governed by the F–N theory, whereas those in the low field are governed by the space-charge-limited current. The Fowler–Nordheim (F–N) equation predicts the current density (*J*) generated from a small surface area of metal based on the electric field (E):

(1) $$J=k_{1}\frac{E^{2}\beta^{2}}{\phi }\cdot \exp\left(-k_{2}\frac{\phi^{1.5}}{\beta \;E}\right),$$

where *k*_1_ = 1.54 × 10^−6^ A·eV/V^2^; *k*_2_ = 6.83 × 10^9^ (V/m)·eV^1.5^ are constants [20]; field enhancement factor *β* is related to geometric design, materials, and usage [25]; *ϕ* is work function of the metals. However, the electric field for the current coplanar electrodes (E to C) is not uniformly distributed. The metal electrode corner near the dielectric exhibits the highest electric field from the modeling [24]. Thus, the distance of the electric takes the shortest distance of the field emission. Figure 4a shows the metals with a lower work function that also exhibit a higher current density (*J*). Figure 4b presents the distance that significantly influences the *J* because the short transport distance renders a strong E.

### 3.2. Finite Element Modeling on Vertical FE Transistors

**Figure 5:**
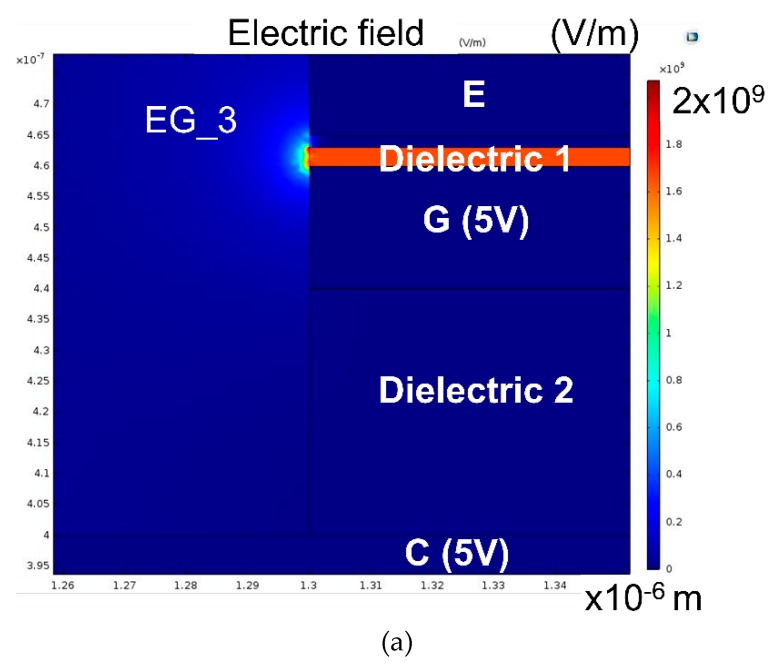

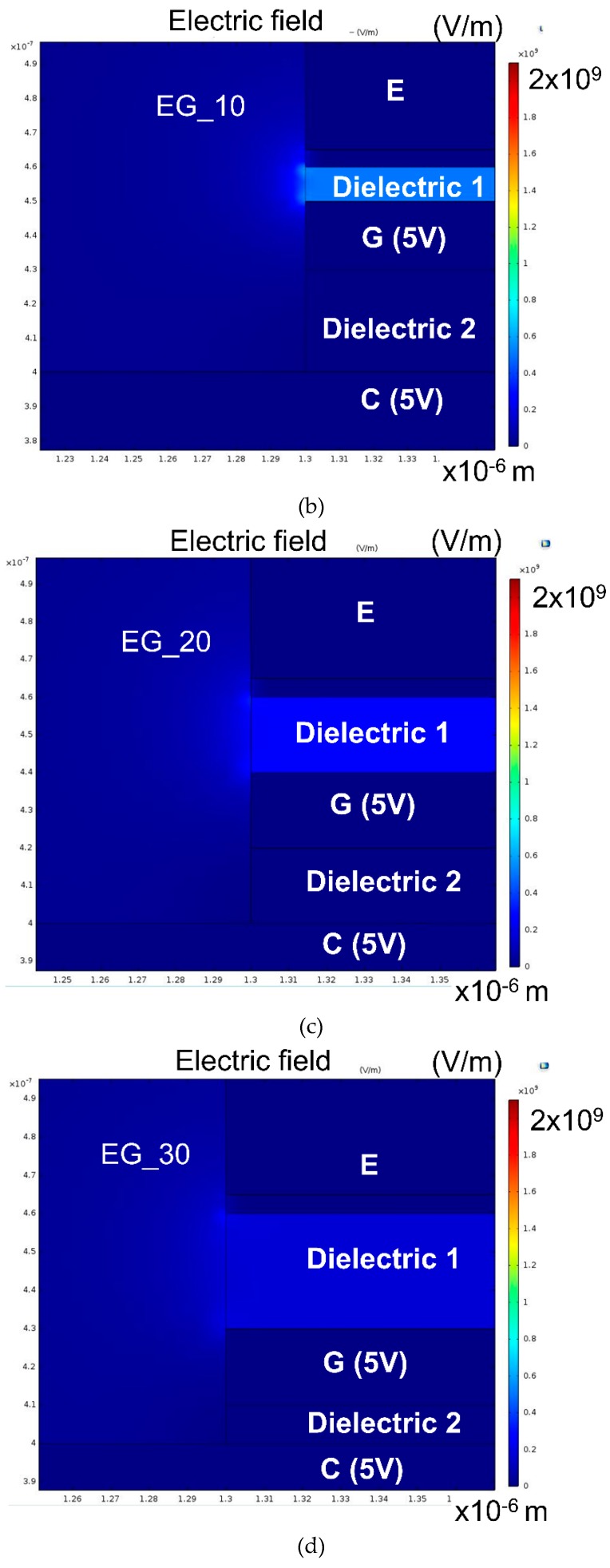
Colors to present electrical fields for (**a**) CG_3, (**b**) CG_10, (**c**) CG_20, and (**d**) CG_30, with V_G_ = V_C_ = 5 V. The color bars are scaled on the same degree.

**Figure 6:**
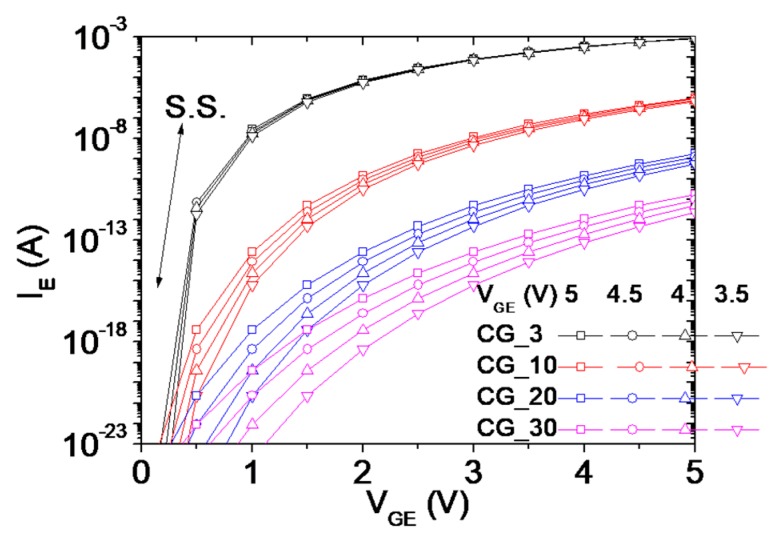
Simulated electric current (I_E_) as a function of voltage (V_GE_) for V_CE_ = 5, 4.5, 4, 3.5 V for CG_3, CG_10, CG_20, and CG_30.

The optimal position located between an emitter and gate was investigated by applying the distribution of electric fields with different (E-to-G) distances of 3 nm (EG_3), 10 nm (EG_10), 20 nm (EG_20), and 30 nm (EG_30), with V_G_ = V_C_ = 5 V grounding the emitter, as presented in Figure 5a–d, respectively. The color scales of the four figures are unified, so the colors in the simulation correspond to the values in the figures. The significant difference in the electric field among the figures occurred only on Dielectric 1 and the air between the gate and emitter, whereas the electric field near Dielectric 2 and the surrounding area makes little difference because the electric field is the gradient of potential difference. Dielectric 1 and its vicinity bear a high difference between its emitter and gate, whereas Dielectric 2 and its vicinity bear a small difference. The EG_3 exhibits the highest electric field near Dielectric 1 among the modeled devices. This result implies that the electric field and current density can be substantial at a small (Dielectric 1) thickness. The electric field distribution is consistent with the finding that the sharp edge of an emitter and collector delivers a high-density emission current [5]. This sharp emitter can also lead to the lowering of threshold voltage from non F–N to F–N behavior [13]. Figure 6 presents the electric current (I_E_) as a function of V_GE_ for the four transistors. EG_3 exhibits the highest I_E_ and the steepest slope, i.e., the smallest subthreshold slope. The modeling results preliminarily conclude that the gate of a vertical transistor should be close to the emitter, i.e., Dielectric 1 should be as thin as possible, to obtain a high-drive current and low current leakage.

**Figure 7:**
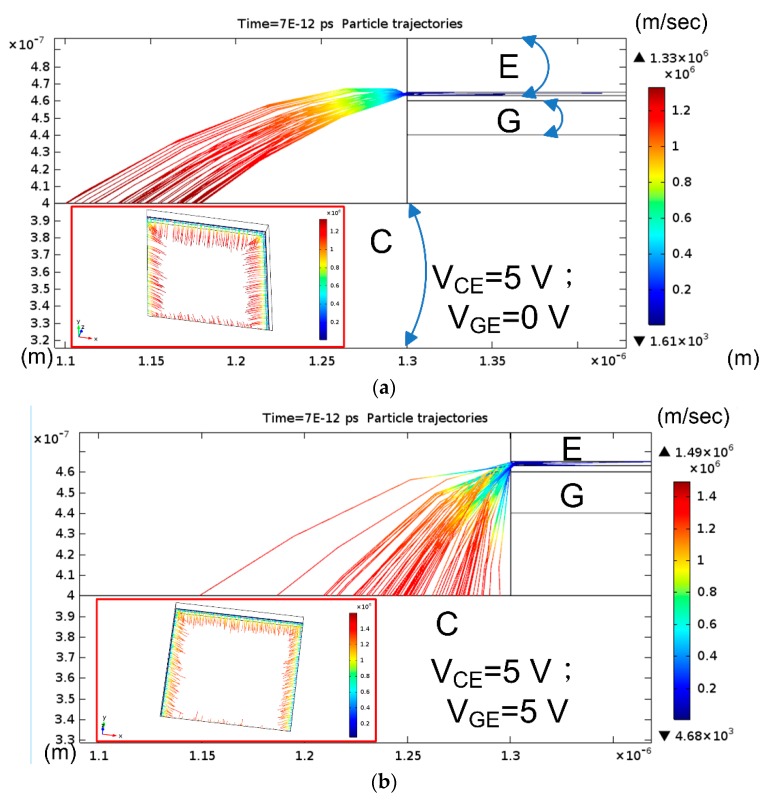
Tracing modeling of the electron trajectories of cross-sectional and three dimentional views (inset) for CG_3 with application of (**a**) V_CE_ = 5 V; V_GE_ = 0 V (**b**) V_CE_ = 5 V; V_GE_ = 5 V.

The particle trajectory by COMSOL can present the tracing route by using the Monte Carlo scattering model. The electrons are accelerated and their final speeds increase with the tracing distance. Given that the metal edge renders the highest electric field [24], this simulation assumes that electrons are ejected from an area limited to 1 nm at the edge of the emitter. A finite number of electrons are projected from the geometric boundary of the emitter. The tracing routes of the electrons shift with gate bias. The cross-sectional view of the model (EG_3) shows that the electrons are accelerated because of the presence of an electron field when the electric potential on the collector (V_CE_) is applied (Figure 7a). Thus, the speeds of the electrons increase along with tracing routes, as indicated by the colors. The bottom collector of the well gathers electrons in the 3D graph (inset of Figure 7a). However, the tracing routes of the electrons are bent down with the application of gate potential (V_GE_) (Figure 7b). The 3D view shows that the tracing routes are short (inset of Figure 7b) and that the final speeds are smaller than those observed without gate bias (V_GE_).

**Figure 8:**
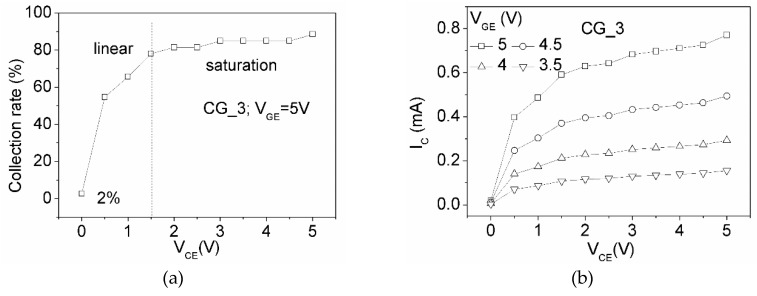
(**a**) Modeling the collection rate of emitted electrons of CG_3 at V_GE_ = 5 V and (**b**) collected electric current (I_C_) at V_CG_ = 3.5, 4, 4.5, or 5 V.

The electric current is counted from a limited number of electrons from the emitter and those accumulated on the collector. The collection of electrons is influenced by V_CE_ and V_GE_. The collection rate at V_CE_ of 0 is only 2% when the V_GE_ = 5 V (Figure 8a). The collection rate significantly increases with V_CE_ (<1.5 V) and saturates when V_CE_ is higher than 1.5 V. The electron flow related to its gate-to-collector electric field is similar to the operation of MOSFETs. The simulation shows that gate potential can substantially enhance the electron flow, similarly to field emission devices [16,17,18,19,20] (Figure 8b). For example, the collector electric current at V_GE_ = 5 V is about five times of that at V_GE_ of 3.5 V. This result indicates that the eliminated gate dielectric of the current vertical field emission devices can perform like typical MOSFETs.

### 3.3. Measurement of Field Emission Vertical Air Channel Diodes

**Figure 9:**
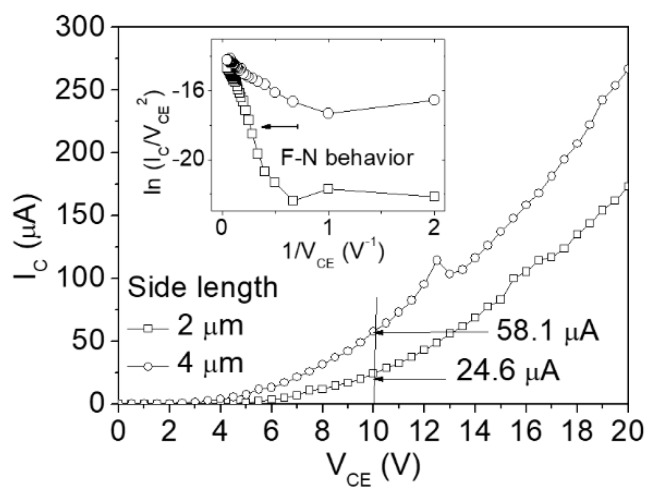
Current–voltage plots of the air channel diodes whose side lengths of the wells are 4 and 2 μm.

The vertical air channel diodes use the top Al-Si-Cu alloy thin film around the well as the emitter. Thus, the emission zone is proportional to the side length. Although the electric field may not evenly distribute on the metal, the measured current is still roughly proportional to the metal area (Figure 9). For example, the measured electrical current for the well with a 4 μm side length is 58.1 μA, whereas that of a well with a 2 μm side length is 24.6 μA at 10 V. Both of the curves exhibit F–N behavior in high electric fields (low 1/V_CE_) and non F–N behavior in low electric fields (high 1/V_CE_) (inset of Figure 9).

The mechanism of tunneling current is described by the F–N plot under high electric field. Based on Equation (1), the negative slope of $\ln(I_{C}/V_{CE})$ as a function of $1/V_{CE}$ is proportional to the emission distance (*d*) (Equation (2)), and the slope of F–N plot is as follows:

(2) $$slope\;(F.N)=-\frac{k_{2}\phi^{1.5}d}{\beta },$$

**Figure 10:**
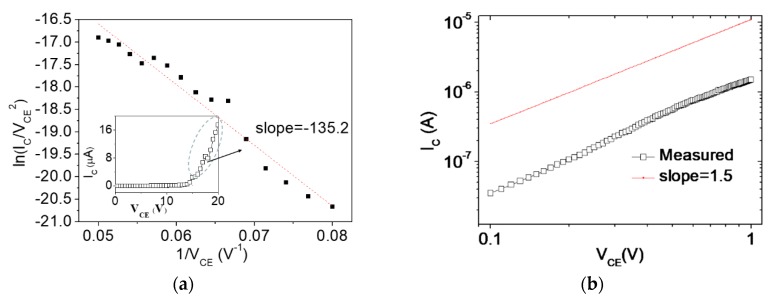
(**a**) F–N plot of the high electric potential part (V_CE_) range where the slope is −135.2 by the regression method and linear scale of current–voltage plot (inset), and (**b**) logarithm current–voltage plot possessing the characteristic of three-half power on a low electric potential part (V_CE_) range governed by a space-charge limited current.

Although the slope can determine the work function of the metals [26], *β* reaches a nonlinear value with increasing $V_{CE}$. When *d* = 60 nm, *ϕ* = 4.28 eV, and the slope is −135.2 by the regression analysis (Figure 10a), the calculated *β* is approximately 26.8. The linear scale of current–voltage exhibits an exponential relationship (inset of Figure 10a) and is similar to the theory presented in Figure 4a,b. By contrast, the space charge limit current equation can be used in FE at a low electric field [27,28]. The predicted current density (*J*) considers the following space charge effect:

(3) $$J=\frac{I_{E}}{S}=\frac{4\varepsilon_{0}}{9}\sqrt{2\frac{e}{m_{e}}}(\frac{V_{E}{}^{3/2}}{d^{2}}),$$

where I_E_ and *S* are the emitted current and the surface inner area, respectively. The *e* and *m_e_* are the magnitude of charge and the mass of the electron, respectively. *d* is the transport distance. The measurement exhibits that the collecting current (I_C_) is aligned with the three-halves power law related to applied voltage (Figure 10b).

**Figure 11:**
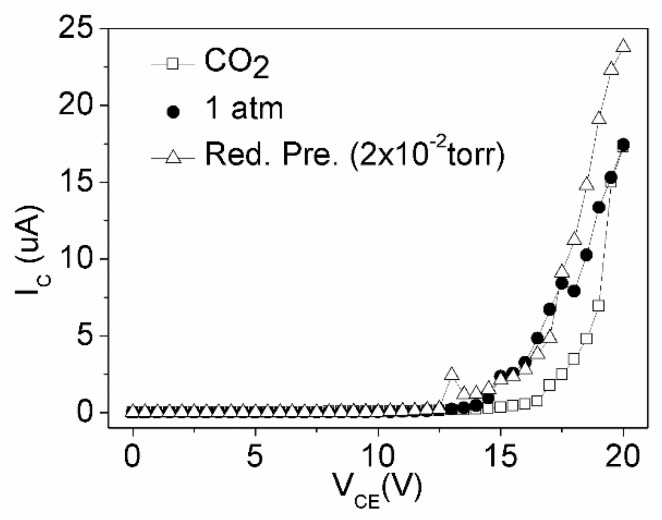
Current–voltage plots for a vertical channel diode with filling CO_2_, atmospheric pressure, and reduced pressure of 10 m-torr.

The proposed device was measured in the closed chamber (Figure 2) for the evaluation of the impact of the collision of the electrons emitted with ambient gases. The chamber was pumped down to a base pressure and fed with CO_2_ from a gas inlet. Then, changes in electric current due to carrier scattering at different ambient environments were evaluated. The measured I_C_ with fed CO_2_ is obviously lower than that under reduced and ambient pressure (Figure 11). This result is consistent with the MFP theory that the collision of field-emitted electrons may differ at different ambient pressure levels and sizes of molecules. The emitted electrons under reduced pressure have less probability of scattering than those in environments with filled CO_2_ and atmospheric pressure. Therefore, a high electric current (I_C_) is collected from the measurement. This result suggests that current emission distance is critical to the sensing of pressure change and molecular size in air because microscale FE devices typically require vacuum environments to operate [16,17,18,19,20]. For example, the MFP ranges from 0.1 mm to 100 mm at a pressure under 0.8–800 m-Torr. The distance can be extremely long for a miniature device and renders tracing routes difficult to control.

## 4. Conclusions

Modeling on air channel FE diodes shows that current density increases when metals of low work function are used and substantially increases with emission distance. The particle trajectory model indicates that the thickness of a gate dielectric should be eliminated in the design of a vertical transistor so that high drive current and low current leakage can be achieved. The behavior of the current–voltage plots related to the application of gate potential is similar to typical MOSFETs from the particle trajectory model. This phenomenon occurs because the transistor, with a short emitter-to-gate distance, exhibits better control on electron emission and a strong electric field near the gate. Although the implementation of a vertical transistor may be limited by the thicknesses of stacked thin films and carrier scattering in air, vertical diodes are fabricated to empirically verify the impact of carrier scattering in air. The measured vertical air channel diodes show that field emission distance (~60 nm that is near MFP) is sensitive to ambient gases and pressure. The measurement results roughly meet the theory of F–N and space-charge-limited current for high and low electric fields; therefore, this current design may be utilized for gas and pressure sensors.

## Figures and Tables

**Table 1:** Four simulated transistors noted by EG_3, EG_10, EG_20, and EG_30, whose transport distance of the channel (D_Channel_) is the sum of the thickness of emitter-to-gate (t_E-G_), gate (t_G_), and gate-to-collector (t_G-C_).

Dimensions of Transistors	EG_3	EG_10	EG_20	EG_30
D_Channel_ (nm)	63	60	60	60
t_E-G_ (nm)	3	10	20	30
t_G_ (nm)	20	20	20	20
t_G-C_ (nm)	40	30	20	10
